# Biologically templated assembly of hybrid semiconducting nanomesh for high performance field effect transistors and sensors

**DOI:** 10.1038/srep35591

**Published:** 2016-10-20

**Authors:** Hye-Hyeon Byeon, Seung-Woo Lee, Eun-Hee Lee, Woong Kim, Hyunjung Yi

**Affiliations:** 1 Post-Silicon Semiconductor Institute, Korea Institute of Science and Technology, Seoul, 02792, Republic of Korea; 2 Department of Nano Semiconductor Engineering, Korea University, Seoul, 02841, Republic of Korea; 3 Department of Environmental Science and Engineering, Ewha Womans University, Seoul, 03760, Republic of Korea; 4 Department of Materials Science and Engineering, Korea University, Seoul, 02841, Republic of Korea

## Abstract

Delicately assembled composites of semiconducting nanomaterials and biological materials provide an attractive interface for emerging applications, such as chemical/biological sensors, wearable health monitoring devices, and therapeutic agent releasing devices. The nanostructure of composites as a channel and a sensing material plays a critical role in the performance of field effect transistors (FETs). Therefore, it is highly desirable to prepare elaborate composite that can allow the fabrication of high performance FETs and also provide high sensitivity and selectivity in detecting specific chemical/biological targets. In this work, we demonstrate that high performance FETs can be fabricated with a hydrodynamically assembled composite, a semiconducting nanomesh, of semiconducting single-walled carbon nanotubes (S-SWNTs) and a genetically engineered M13 phage to show strong binding affinity toward SWNTs. The semiconducting nanomesh enables a high on/off ratio (~10^4^) of FETs. We also show that the threshold voltage and the channel current of the nanomesh FETs are sensitive to the change of the M13 phage surface charge. This biological gate effect of the phage enables the detection of biologically important molecules such as dopamine and bisphenol A using nanomesh-based FETs. Our results provide a new insight for the preparation of composite material platform for highly controllable bio/electronics interfaces.

Integration of semiconducting nanomaterials with biological materials has been extensively investigated in various fields including bio-sensors, brain science, cancer research, tissue engineering, and drug delivery^1^,^2^,^3^,^4^,^5^. The integration has been motivated and driven by the advantages of the semiconducting nanomaterial-based devices that are sensitive to small amount of chemical or biological targets and/or the nanostructures that provide large effective surface area for maximal interaction with ionic environment^6^,^7^,^8^,^9^. However, the device performance strongly depends on the dispersion state of individual materials in the nano/bio composites and the amount of defects generated during the fabrication process^10^,^11^,^12^. Therefore, the challenge is to prepare the nano-bio composite materials in which each nanomaterial is effectively separated from one another without being agglomerated while intrinsic properties of the nanomaterials and the biomaterials are well-preserved during the dispersion process which would contribute to maximizing device performance^13^,^14^.

Molecular recognition of biological materials not only enables selective binding to target chemical or biological species but also offers a non-destructive means to precisely control the interactions between biomolecules and non-biological materials^15^,^16^,^17^,^18^. Various biomolecules such as DNA, peptides, and proteins have been utilized to functionalize surfaces of electronic materials or to stabilize nanoscale electronic materials in aqueous solution without deteriorating their electronic properties^19^,^20^,^21^,^22^. For example, specific short peptide sequences have been identified through affinity-based selection processes such as bio-panning and have been utilized for binding of various electronic materials^17^,^23^,^24^,^25^,^26^. In addition, biological materials inherently possess various chemical functional groups. Therefore, a biological template material encoded with specific binding affinity toward nanoscale electronic materials could serve as an ideal platform to assemble nanoscale semiconducting material and at the same time confer additional functionality for highly controllable electrochemical interfaces.

Recently, it has been demonstrated that a filamentous M13 phage showing strong binding affinity to the surface of single-walled carbon nanotubes (SWNTs) could effectively disperse unsorted SWNTs and can be used to assemble a conductive nanomesh *via* a hydrodynamic assembly process^27^,^28^. An M13 phage is a filamentous biological material with a diameter of ~6.5 nm and a length of ~880 nm, and the capsid proteins at various locations of the M13 phage can be genetically engineered to include specific peptide sequences that bind targeted materials^29^,^30^,^31^. Owing to its filamentous shape, the M13 phage has been shown to effectively interact with one-dimensional nanomaterials such as SWNTs^32^. The assembled conductive nanomesh had fine nanostructures, high conductivity and good adhesion with various substrates including flexible substrates. As a result, the conductive nanomesh-integrated flexible neural probes drastically decreased the *in vivo* contact impedance between bio-fluids and the neural probes, and significantly improved the detection rate of biologically important but weak brain signals of mouse^27^. However, applications of the conductive nanomesh are limited due to very low on/off current ratio (I_on_/I_off_ ~ 10); successful assembly of semiconducting nanomesh would greatly extend the scope of applications towards the fabrication high performance transistors and biological sensors.

Here, we demonstrate that high quality semiconducting nanomesh can be fabricated based on the genetically engineered filamentous biological template, M13 phage, and semiconducting-enriched SWNTs (S-SWNTs). We also report a biological gate effect of the semiconducting nanomesh-based FETs. The semiconducting nanomesh can be prepared in macroscale (~several centimeters) and an array of nanomesh-based electronic devices can be fabricated without relying on lithographic, chemical or annealing process. Owing to the excellent dispersion of the S-SWNTs, nanomesh FETs show high on/off ratio of 10^4^. Importantly, the semiconducting nanomesh-based FETs are sensitive to the change of the local charge density and pH *via* the biological gate effect of the phage. This charge sensitive nature allows the semiconducting nanomesh-based electrolyte-gated FETs (e-FETs) to detect important biological chemicals such as dopamine and bisphenol A. We envision that the biologically integrated semiconducting nanomesh would serve as a new material platform for highly controllable bio/electronics interfaces and flexible biosensors.

## Results

### Biologically templated assembly of semiconducting nanomesh

**Figure 1:**
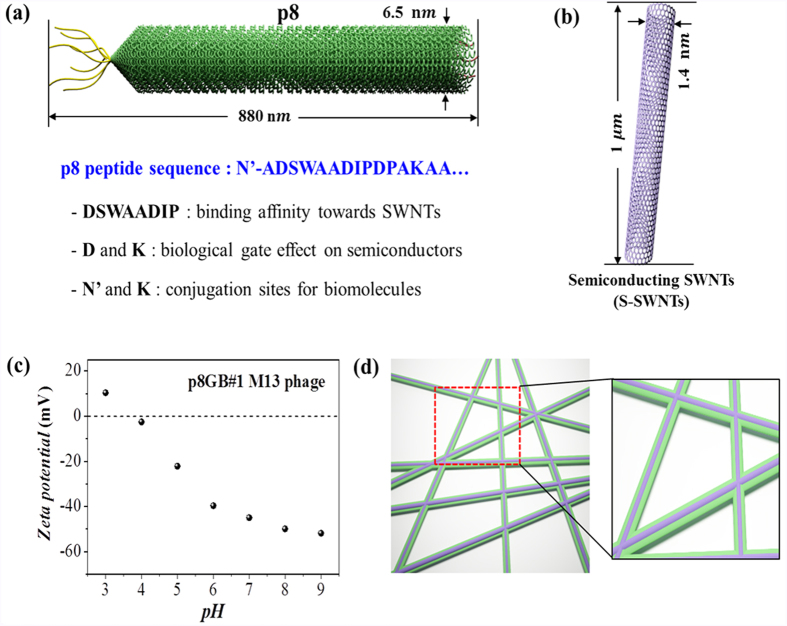
Components and the nanostructure of the semiconducting nanomesh. (**a**) A schematic of the biological template material, p8GB#1 M13 phage and (**b**) the semiconducting SWNT (S-SWNT). The phage contains a specific peptide sequence for binding SWNTs expressed on its body surface (p8). The body surface peptides also function as a biological gate on S-SWNTs and provide conjugation sites for additional biomolecules. (**c**) Zeta potentials of the p8GB#1 phage at various pH values. (**d**) A schematic of the nanostructure of the semiconducting nanomesh. The M13 phage also serves as a biological gate on the intimately bound S-SWNTs *via* the charged surface peptides.

Figure 1a schematically illustrates the biological template material, p8GB#1 M13 phage containing on its surface (p8) a specific peptide sequence with strong affinity for SWNTs, to bind and to align S-SWNTs (Fig. 1b) along the length of its body. The peptide sequence of the p8GB#1 M13 phage was identified by applying a bio-panning process using a phage display p8 peptide library that was constructed in our laboratory^28^. The hydrophobicity pattern and the aromatic amino acid tryptophan (W) of p8GB#1 are expected to be mainly responsible for the interaction with S-SWNTs^27^. The surface peptide of the p8GB#1 phage has also various charged functional groups, including acidic (D, carboxyl group, pK_a_ ~ 3.9) and basic (K, amine, pK_a_ ~ 10.54) residues, as shown in Fig. 1a and Fig. S1. The net surface charge of the phage produces an effective electric field in the electrolyte solution according to Poisson equation^33^. The net surface charge density can be inferred from the zeta potential, and the measured zeta potential of the p8GB#1 phage is presented in Fig. 1c. Since the pK_a_ of the side chain functional group of D is lower than that of K, the change of the net surface charge was dominated by deprotonation of the carboxyl group as the pH was increased within the tested pH range. Since potential (electric field) is directly related to the surface charge density and the M13 phage is directly interfacing with the semiconducting SWNT- channel, the M13 phage can behave as local gate that is sensitive to local charge density *via* change of pH or binding of small molecules, as schematically illustrated in Fig. 1d and demonstrated further below^33^.

**Figure 2:**
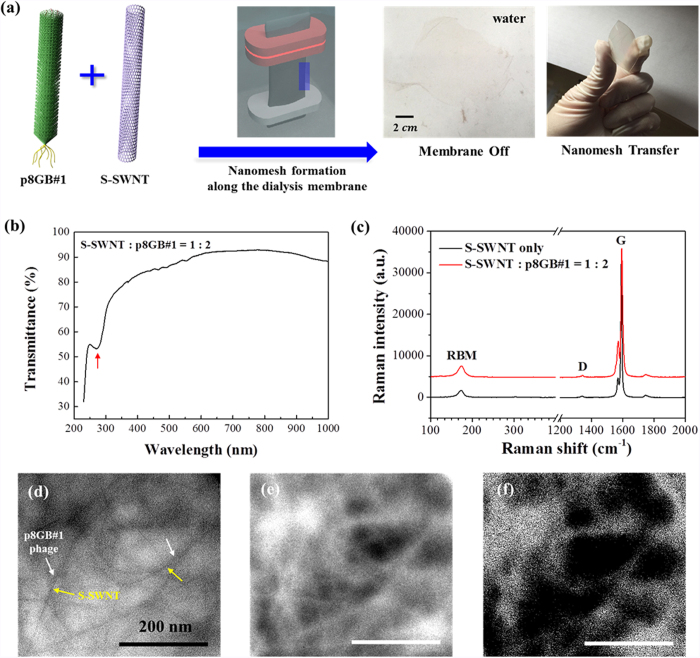
Fabrication processes and characteristics of the semiconducting nanomesh. (**a**) Processes to assemble hybrid semiconducting nanomesh using a biological template material in solution and the processes to provide the semiconducting nanomesh on arbitrary substrates. The semiconducting nanomesh transferred onto a transparent flexible substrate (PES) is shown. (**b**) The transmittance curve of the nanomesh. The molar ratio of S-SWNT:p8GB#1 = 1:2. The absorption peaks in the wavelength range of 400 nm ~600 nm correspond to the optical transition peaks from S-SWNTs and the large absorption peak around 270 nm is due to the M13 phage^30^,^32^. (**c**) Raman spectrum of the hybrid semiconducting nanomesh on a SiO_2_/Si substrate compared with the solution-dispersed S-SWNTs drop-casted on a SiO_2_/Si substrate (S-SWNT-only). (**d**) High-magnification transmission electron micrograph of the assembled hybrid semiconducting nanomesh. The sharp and high-contrast lines (indicated by yellow arrows) in the image correspond to the S-SWNTs whereas the dark and diffuse lines (indicated by white arrows) overlapping with the S-SWNTs are from the p8GB#1 phage. (**e**) Carbon mapping image. (**f**) Nitrogen mapping image. Since the M13 phage contains both carbon and nitrogen whereas SWNTs possess carbon only, the nitrogen mapping indicates the locations of the M13 phage.

Figure 2a schematically illustrates the process to assemble semiconducting nanomesh. The hydrodynamic assembly works based on a concentration polarization effect^27^. Concentration polarization is a diffusion-based hydrodynamic phenomenon in which the concentration of colloidal particles such as S-SWNTs and the M13 phage that are bigger than the molecular-weight cut-off (MWCO) of the dialysis membrane become enriched whereas species smaller than the MWCO of the membrane such as surfactant molecules and ions become depleted near the inside boundary of the dialysis membrane. When surfactant-dispersed S-SWNTs were dialyzed in the presence of p8GB#1 phage, p8GB#1 phage bound S-SWNTs and the p8GB#1-bound S-SWNTs were assembled into a nanostructured network, i.e., a nanomesh around the dialysis membrane. Here, it is noted that the sorted S-SWNTs were surfactant-exchanged with sodium cholate before the hydrodynamic process for the stable assembly of the nanomesh. Removing the dialysis membrane after the dialysis readily produced a large freestanding nanomesh film in solution as shown Fig. 2a. In contrast, in the absence of p8GB#1, S-SWNTs formed fragile films or aggregates and did not form large stable films (Fig. S2). Moreover, a different M13 phage clone that did not have binding affinity toward S-SWNTs also only produced aggregates or fragmented SWNTs (Fig. S3). These results imply that the binding affinity of p8GB#1 phage is critical for the formation of the nanomesh. It also suggests that sorted S-SWNTs can be successfully assembled into a nanomesh using the hydrodynamic assembly method *via* surfactant exchange process. The thickness of the semiconducting nanomesh film produced in the presence of p8GB#1 was about ~100 nm and the size was ~100 cm^2^. The size of the nanomesh, being dependent on the size of the dialysis membrane, could be in principle further scaled up by using larger dialysis membranes. The isolated freestanding nanomesh was readily transferred onto various substrates without chemical etching as shown in Fig. 2a. The transmittance curve of the nanomesh transferred onto a transparent substrate is shown in the Fig. 2b. The molar ratio is S-SWNT:p8GB#1 = 1:2. Here, it is noted that although the size of the M13 phage is much bigger than the S-SWNTs, allowing binding of multiple SWNTs per M13 phage, a compatible molar ratio of the M13 phage to the S-SWNTs was used due to the slower diffusion of the M13 phage than the SWNTs during the nanomesh assembly^27^. The absorption peaks in the wavelength range of 400 nm ~600 nm correspond to the optical transition peaks from the S-SWNTs and the large absorption peak around 270 nm is due to the M13 phage^30^,^32^.

The non-destructive nature of the biological template-based SWNT-assembly and the transfer process was confirmed using Raman spectroscopy^34^. The Raman spectrum of the nanomesh transferred onto a SiO_2_/Si substrate is shown and compared with the one from the as-received S-SWNTs in Fig. 2c. The nanomesh yielded a radial breathing mode (RBM) peak that is a characteristic of carbon nanotubes, the graphitic peak (G), and a defect-related peak (D, ~1,400 cm^−1^) in the spectrum. The negligible intensity of the D peak and the similarity of this Raman spectrum to that from the as-received S-SWNTs confirmed the non-destructive nature of the assembly and the transfer process. The nanostructure and the binding scheme of the assembled semiconducting nanomesh were examined using transmission electron microscopy (TEM). The transmission electron micrograph and the energy-filtered (EF) mapping of the corresponding region of the semiconducting nanomesh are shown in Fig. 2d–f. In general, SWNTs produce sharp and high-contrast lines whereas the biological materials such as M13 phage show dark and diffused lines. The transmission electron micrograph of the semiconducting nanomesh shows sharp lines (indicated by yellow arrows) overlapped with dark and diffuse lines (indicated by white arrows), suggesting the p8GB#1 phage bound S-SWNTs along the body lengths in the nanomesh. To further confirm the binding scheme of the semiconducting nanomesh, an element specific TEM mapping was conducted. The carbon and the nitrogen mapping images of the corresponding TEM image are shown in Fig. 2e,f, respectively. Since S-SWNTs and surfactants do not have nitrogen elements whereas the p8GB#1 M13 phage contains nitrogen components coming from the peptides, the element specific TEM confirms that the p8GB#1 phage bound the S-SWNTs along its body length within the nanomesh matrix, as schematically illustrated in Fig. 1d. This binding scheme is similar to the conductive nanomesh assembled using mixed SWNTs^27^. However, when using the semiconducting-enriched SWNTs, this unique binding scheme of the hybrid nanomesh and the non-destructive assembly process additionally enabled biological gating by the phage on the semiconducting SWNTs, as demonstrated further below.

### Semiconducting nanomesh-based FETs

**Figure 3:**
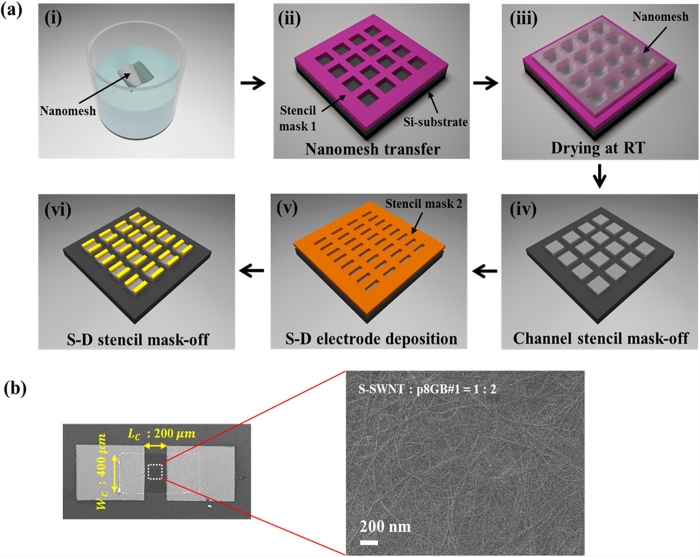
Non-destructive fabrication of semiconducting nanomesh-based FETs. (**a**) Processes used to fabricate the semiconducting nanomesh-based back-gated FET. (**b**) The scanning electron micrographs of a representative back-gated FET device and the channel region. The molar ratio of S-SWNT:p8GB#1 = 1:2.

**Figure 4:**
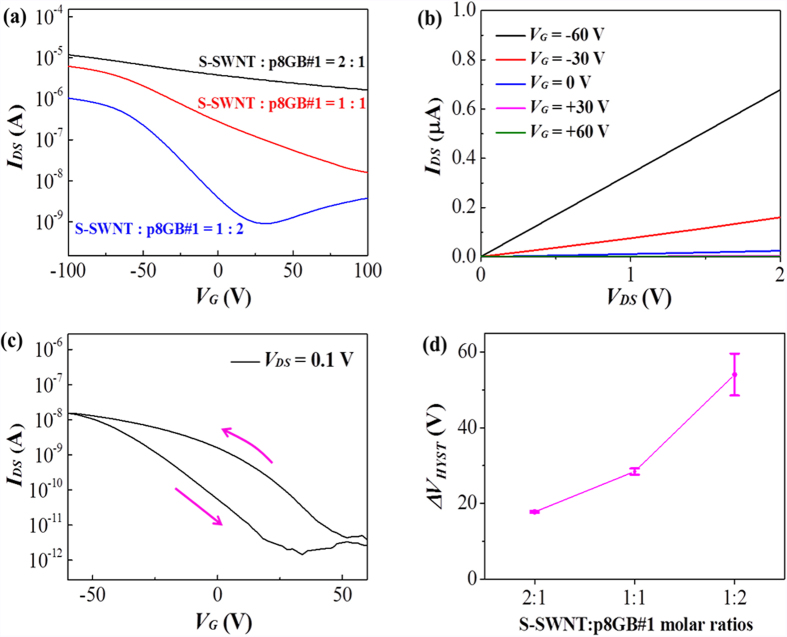
Characteristics of the semiconducting nanomesh-based FETs. (**a**) The effect of the phage on the transfer characteristics of the semiconducting nanomesh-based FETs. The (**b**) output characteristics and (**c**) transfer characteristics of the nanomesh-based FET with the molar ratio of 1:2. (**d**) The effect of the phage on the hysteresis of back-gated FETs. The ΔV_HYST_ is defined as the voltage width at the half-maximum of I_DS_. Each data point is the mean ± s.d. from n = 3 devices.

The electrical transport properties of the semiconducting nanomesh as an FET channel have been investigated by controlling the molar ratio of S-SWNT:p8GB#1 to produce a high I_on_/I_off_ of macroscale field-effect transistors. The processes to fabricate the FETs are schematically illustrated in Fig. 3a. The scanning electron micrographs of a representative FET device and the channel region are shown in Fig. 3b. The transfer characteristics (the source-drain current, I_DS_, versus the gate voltage, V_G_) and the output characteristics (I_DS_ vs. the source-drain voltage, V_DS_) from the nanomeshes of different molar ratios are presented in Fig. 4a and Fig. S4. These curves show that as the molar ratio of the phage was increased, the overall I_on_/I_off_ drastically improved. Here, the I_on_/I_off_ was defined as the difference between the maximum and the minimum I_DS_ for an applied V_DS_. The increased I_on_/I_off_ with increasing molar ratio of p8GB#1 is mainly due to the decreased I_off_. The I_off_ is generally related to the metallic SWNTs^10^. Although we used 99% semiconducting enriched SWNTs, there still exist metallic SWNTs. In addition, the nanomesh with the higher molar ratio of the phage showed more effectively de-bundled networks (Fig. S5). Aggregated S-SWNTs could transfer electrons between tubes^32^. When the bandgap of S-SWNTs is small (when the diameter of SWNTs is large) and the heterogeneity of dimensions of SWNTs is large, the aggregated S-SWNTs could behave as metallic ones, leading to a high I_off_ and accordingly lower I_on_/I_off_. Note that the slightly reduced I_on_ with increasing molar ratio of the phage is due to the electrically insulating nature of the phage. Therefore, these results suggest that the p8GB#1 phage improved the overall device performance by preventing S-SWNTs from bundling and by providing well-controlled nanostructures over a large area.

The output characteristics and the transfer characteristics of a representative back-gated FET from the 1:2 nanomesh are shown in Fig. 4b,c. The best I_on_/I_off_ was found to be ~1.34 × 10^4^ at V_DS_ ~ 0.1 V. The I_on_ and I_off_ values were observed to be 1.61 × 10^−8^ A and 1.20 × 10^−12^ A, respectively. The threshold voltage, V_th_, was −23.83 V, with a hole mobility, μ_h_, of 0.17 cm^2^/V s at V_DS_ = 0.1 V (supplementary equation). The I_on_/I_off_ ratio of the semiconducting nanomesh is much higher than that of the nanomesh from mixed SWNTs by three orders of magnitude^27^. Although this value is lower than the reported highest performance from SWNT-based FETs realized using other approaches, the performance of the nanomesh-based FET could be still compatible to those from high-performance SWNT-FETs as summarized in Fig. S6. These results suggest that the biologically templated assembly approach can provide macroscale high-performance SWNT-based FETs at a potentially large scale under ambient conditions in a simple and non-destructive manner.

Note that the transfer curve of the nanomesh showed anticlockwise hysteresis as indicated by the arrows of Fig. 4c. The degree of hysteresis, ΔV_HYST_, defined as the voltage width at the half-maximum of I_DS_, increased with increasing molar ratio of the phage as shown in Fig. 4d. It is nown that the water molecules and the silanol groups on the surface of the SiO_2_ back-gate oxide are largely responsible for the hysteresis of the back-gated CNT FET^35^,^36^,^37^. Due to the many hydrophilic amino acid residues (D, S, and K) on the surface of the p8GB#1 phage body (Fig. 1a and Fig. S1), the increasing hysteresis of the semiconducting nanomesh was ascribed to the increased hydrophilicity of the assembled nanomesh. However, this hysteresis became negligible under an electrolyte gated configuration as demonstrated below. This could be ascribed to the back-gate dielectric, which is mainly responsible for hysteresis, not having been involved in the electrolyte gated configuration^38^,^39^.

### Biological gate effect of the semiconducting nanomesh-based e-FETs

**Figure 5:**
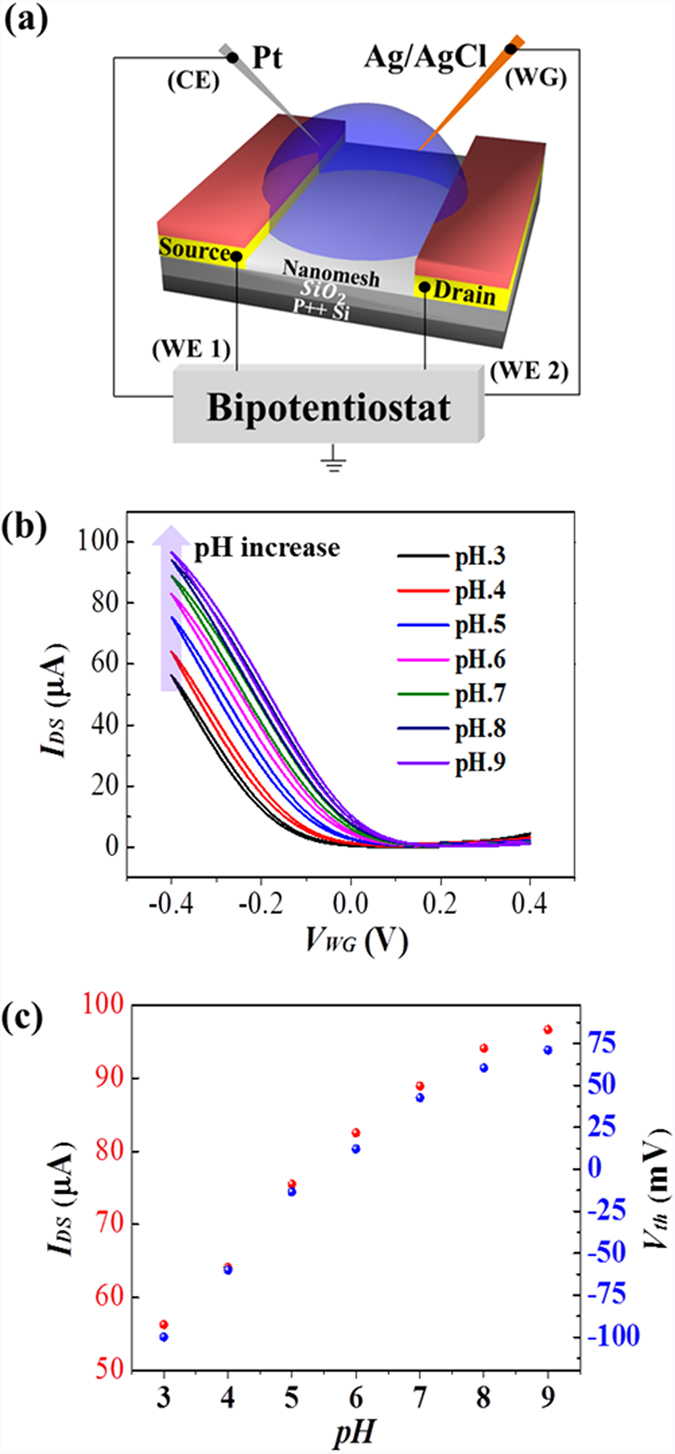
Semiconducting nanomesh-based e-FETs and the biological gate effect of the surface charge of the M13 phage. (**a**) A schematic illustration of the e-FET device and the configuration used to make the measurements. The Ag/AgCl reference electrode serves as the water-based electrolyte gate (WG). (**b**) The transfer characteristics of the e-FET at various pH values. V_DS_ = 0.4 V. (**c**) The dependence of the I_DS_ and the V_th_ of the semiconducting nanomesh-based e-FET on pH.

The biological template approach enables an intimate contact between the chemical functional groups of the phage and the semiconducting SWNTs along their lengths. To investigate the gate effect of the phage template on the semiconducting channel, at first the transfer curves at different pH values were examined under an e-FET configuration, as schematically shown in Fig. 5a^40^. For the e-FET measurement, the source, drain and the water-based electrolyte gate (WG) potential were driven in a biological liquid electrolyte (10 mM PBS) using a bipotentiostat system (Autolab, PGSTAT 128N). The source and drain electrodes were designated as two working electrodes (WE1 and WE2), the Ag/AgCl water-based electrolyte gate (WG) as reference electrode (~2 mm dia. filled with 3 M KCl), and the additional Pt wire as the counter electrode (CE). The output characteristics of the e-FET are shown in Fig. S7. The transfer characteristics of the e-FET measured at various pH conditions are shown in Fig. 5b. Here, it is noted the V_th_ value of the e-FET was found to be less than 1 V, which is significantly lower than the value from the back-gated FET. Under the e-FET scheme, the quantum capacitance of the S-SWNTs, C_SWNTs_, and the electric double-layer (EDL) capacitance, C_EDL_, forming around the Ag/AgCl gate are connected in series, and therefore the C_SWNTs_, being much lower than C_EDL_, dominates the overall capacitance of the device. In addition, the C_SWNTs_ is known to be much higher than the capacitance of back-gate dielectric (SiO_2_), C_300 nm-SiO2_: that is, C_300 nm-SiO2_ ≪ C_SWNTs_ ≪ C_EDL_^38^,^39^. Therefore, the operation voltage of the e-FET became significantly lower than that of the back-gated FET.

Figure 5b shows that as the pH was increased, the transfer curve shifted toward a more positive voltage; that is, the I_DS_ increased at a fixed gate voltage, V_WG_. The V_th_ values and the I_DS_ levels at various pH values are plotted in Fig. 5c. The V_th_ shift was observed to be about 30 mV/pH and the current response was about 6.7 μA/pH. The V_th_ shift can be explained by the change of the surface charge of the p8GB#1 phage in aqueous solution; that is, as the pH was increased, the phage became more negatively charged (Fig. 1c) and hence induced more positive carriers in the channels, shifting the threshold toward a more positive potential, as shown in Fig. 5b,c. Note that, as the pH was increased, the response of the e-FET began to increase more slowly at around pH ~ 7. The flattening behaviour well correlates with the zeta potential dependence of the phage on pH in Fig. 1c, confirming the gate effect of the phage on S-SWNTs. In the nanomesh, the chemical functional groups of the biological template is in intimate contact with the S-SWNTs and therefore the phage serves as an effective gate for the S-SWNTs even in the high ionic strength of buffer solution. Note that the ionic strength of the pH buffer solution was high, at 10 mM. The Debye length, λ, which describes the extent to which the electric field, -∇ψ, extends from the charged surface, decreases with increasing ionic strength (being ~a few nm in 10 mM electrolyte) since the surface charge and the resultant electric field are readily screened by counter ions in solution^33^,^41^. Therefore, only when the sensing channel is within the Debye length, can any change of charge caused by a chemical or biological reaction or by a specific binding event be electrically detected in an effective manner^42^.

**Figure 6:**
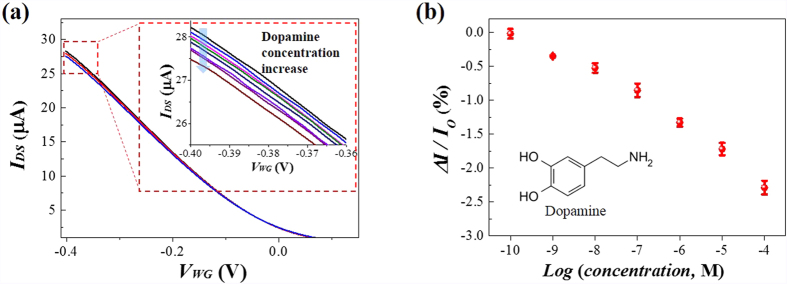
Electrochemical modulation of the semiconducting nanomesh-based e-FET by dopamine binding. (**a**) The transfer characteristics of the semiconducting nanomesh-based e-FET at various concentrations of dopamine in a biologically relevant solution (10 mM PBS buffer solution). Enlarged views of the transfer curves around V_WG_ ~ −0.4 are shown in the inset. (**b**) The dependence of the I_DS_ on the concentration of dopamine. Each data point is the mean ± s.d. from n = 3 devices.

The biological gate effect of the biological phage material was further investigated using dopamine. Dopamine was chosen since it has an aromatic group that is expected to interact with the aromatic residue (W, tryptophan) on the surface of the body of the p8GB#1 *via* π-π interactions^43^; at the same time it is positively charged (pI ~ 8.93) in physiological solution (pH ~ 7.4)^44^ and therefore the binding of dopamine could reduce the effective charge density of the phage. Figure 6a shows the transfer characteristics of the e-FET measured at various concentrations of dopamine in 10 mM PBS buffer solution. The limit of detection (LOD, S/N = 3) for the dopamine was estimated to be ~80 pM. As the concentration of dopamine was increased, the I_DS_ value was found to decrease. The change in current as the dopamine concentration was increased at a V_WG_ of −0.4 V is presented in Fig. 6b. Note that several studies have reported that dopamine introduced a p-doping effect on SWNTs, reduced graphene oxide (rGO), and CVD-graphene-based e-FETs, leading to an increase of I_DS_ or positive shift of V_th_^9^,^45^. This behaviour is in contrast to our results where the V_th_ shifted toward a more negative voltage and the I_DS_ decreased. It seems that in our system the modulation of the gate effect of the phage by the binding of dopamine was more dominant than the doping effect of the adsorbed dopamine on the S-SWNTs, which is ascribed to the preferential binding of the aromatic molecules with the phage and the interaction of the majority of the S-SWNT surface with the bigger phage.

### Selective detection of BPA with aptamer-conjugated nanomesh-based e-FET

**Figure 7:**
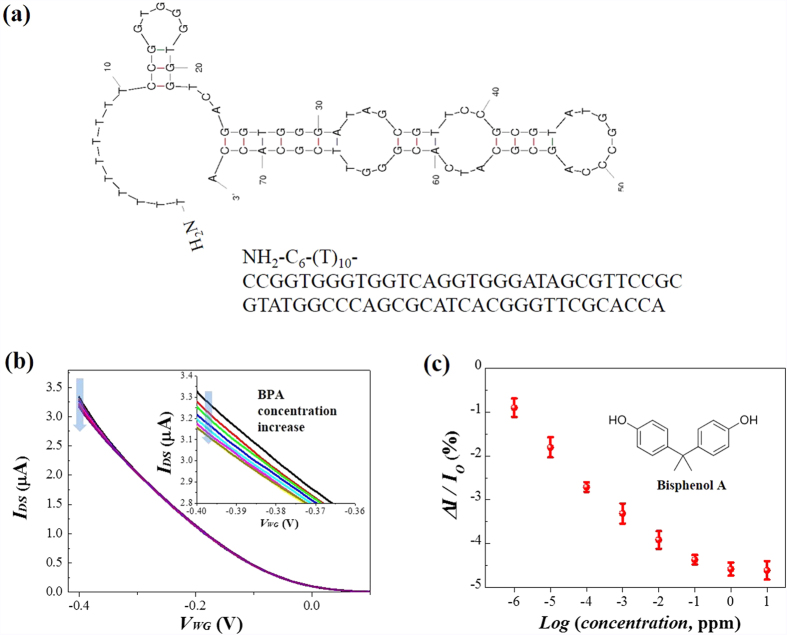
BPA detection with the aptamer-conjugated semiconducting nanomesh-based e-FET. (**a**) Single-stranded DNA aptamer structure for binding of bisphenol A (anti-BPA aptamer). (**b**) The transfer characteristics of the aptamer-conjugated nanomesh-based e-FET at various concentrations of BPA in a biologically relevant solution (10 mM PBS buffer solution). Enlarged views of the transfer curves around V_WG_ ~ −0.4 are shown in the inset. (**c**) The dependence of the I_DS_ on the concentration of BPA. Each data point is the mean ± s.d. from n = 3 devices.

The dominant gating effect of the biological template material on the nanostructured semiconducting channels further allows for additional functionality *via* modification of the template surface. We conjugated an aptamer, a single-stranded DNA or RNA oligonucleotides, onto the phage surface to specifically bind small molecules. In this study, we utilized a specific aptamer designed for bisphenol A (BPA) detection as previously described in literature^46^. We chose BPA since endocrine disruptors such as BPA disrupt the normal endocrine functions influencing sexual development and reproduction of humans and animals, and ultimately result in infertility and various cancers. BPA associated with the cell membrane can simulate physiological responses even at low BPA concentrations (~100 pM)^46^,^47^. The sequence of ssDNA aptamer and its schematic structure is presented in Fig. 7a. Figure 7b shows the transfer characteristics of the anti-BPA aptamer-conjugated nanomesh-based e-FET upon addition of various concentrations of BPA. The limit of detection (LOD, S/N = 3) for the BPA was ~0.4 ppt. The I_DS_ linearly decreases up to 0.1 ppm level of BPA, and starts flattening after 1 ppm of BPA. This could be explained by the decreased effective negative charge of the aptamer anchored on the M13 phage, thus reducing the effective field acting on the S-SWNT channels in intimate contact with the phage in the nanomesh^47^,^48^,^49^,^50^. It has been proposed that the specific aptamer that we employed in this study specifically bound BPA and the binding reduced the effective charges around the aptamer presumably *via* either conformational change of the negatively charged aptamers or the screening of the aptamer by the specifically bound BPA^48^,^49^,^50^. It is noted that this preferential binding-induced response of the e-FET is much lower than the pH–induced current modulation shown in Fig. 5b,c. Unlike the pH induced biological gating effect where the pH directly changes the surface charge of the M13 phage and accordingly the effective field, the binding of biomolecules indirectly affect the field acting on the SWNTs, resulting in a relatively weak response compared to the pH change. However, owing to the intimate contact of the SWNTs with the phage, the e-FET could detect BPA in a buffer solution of a high ionic strength.

We found that the aptamer-conjugated semiconducting nanomesh did not significantly respond to dopamine, or other BPA analogues such as bisphenol B and bisphenol C (Fig. S8) that are not expected to preferentially bind to the anti-BPA aptamer. These results suggest that our approach provides a promising material platform for bio/electronics interfaces.

## Discussion

We have demonstrated a biologically templated assembly of a high quality semiconducting nanomesh. The exquisite assembly of the individual S-SWNTs and M13 phages can lead to the fabrication of high-performance FETs (I_on_/I_off_ ~ 10^4^) without relying on lithographic, chemical or annealing processes. Demonstration of high performance FETs indicates that the S-SWNTs can be effectively dispersed in the nanomesh *via* hydrodynamic process in which bundle formation or defect generation is minimized. We also showed the biological gate effect of the biological template material, M13 phage, on the semiconducting nanomesh. The biological gate effect enabled controllable and selective electrochemical interface modulation of the electronic nanomaterials. The semiconducting nanomesh-based e-FETs could detect BPA at the concentration level of ppt. The nanomesh-e-FET-based sensors could have great potential for detecting analytes in a pH controlled buffer solution or physiological solutions or sensing biological reactions that can change the pH surrounding the nanomesh channels. Our work suggests a promising route to realizing high-performance macroscale semiconductor devices out of nanoscale semiconducting materials and emerging applications such as wearable health monitoring devices and controlled release and monitoring of therapeutic agents^51^,^52^.

## Methods

### Biologically templated assembly of the semiconducting nanomesh

The semiconducting nanomesh was hydrodynamically assembled using a biological template material as shown in Fig. 1a^27^. Briefly, as-received 99% semiconductor-enriched SWNT solution (IsoNanotubes-S-99% from NanoIntegris Inc.) was first dialyzed against a sodium cholate solution (anionic surfactant, 2% w/v in deionized water) for two days using a dialysis membrane (SpectraLabs, product #132706, MWCO 12,000 ~ 14,000 Daltons) with frequent buffer changes. This surfactant-exchange process is critical to effectively debundle and stabilize individual S-SWNTs. Unlike unsorted or mixed SWNTs, the as-received S-SWNTs are coated with surfactants used in the process of enriching or sorting S-SWNTs. This surfactant causes the agglomeration of S-SWNTs in the nanomesh. Proper replacement of it with sodium chlorate leads to high quality semiconducting nanomesh and high performance FETs. The S-SWNTs stabilized by the sodium cholate surfactant were mixed with the M13 phage showing strong binding affinity toward SWNTs on its body surface, p8GB#1 phage, with various S-SWNT:p8GB#1 molar ratios. A 5 × 10^11^/mL number concentration of S-SWNTs was used to prepare a nanomesh with a 1:2 molar ratio of S-SWNT:p8GB#1 (Supplementary equation). The mixed solution was then put into a dialysis membrane (SpectraLabs, product #132700, MWCO 12,000 ~ 14,000 Daltons) and dialyzed against deionized water with frequent changing of the dialyzing solution. After about 30 hours, the dialyzed membrane bag was taken out to a container filled with water and then the dialysis membrane was removed, producing a large-area nanomesh film floating in water.

### Characterizations of the assembled nanomesh

The Raman spectra for the S-SWNTs were obtained using a RENISHAW InVia Raman Microscope, using a 532 nm laser with a focal beam size of 2 μm. For the TEM and EF-TEM measurements, the nanomeshes were transferred to a TEM grid (QUANTIFOIL 2 μm circular holes, TedPella Inc.) and dried at room temperature. TEM and EF-TEM were performed using a Quantum 966 of FEI Titan, operated at 300 kV.

### Zeta potential measurement

For measuring the zeta potential of p8GB#1 phage, seven different phosphate citrate buffer solutions (1 mM, pH values from 3 to 9) were prepared by mixing 150 μL of p8GB#1 phage (1.4 × 10^14 ^phage/ml, ~4 mg/ml) with 600 μL of 1 mM phosphate citrate buffer. The zeta potential of p8GB#1 phage in phosphate citrate buffer solution was measured using a Malvern Zetasizer Nano ZS.

### Fabrication of semiconducting nanomesh-based FETs and e-FETs

Freestanding nanomesh was transferred onto a cleaned SiO_2_ (300 nm)/Si substrate using a pre-patterned stencil mask. The transferred layer was left to dry in air, and then the stencil mask was lifted off to produce channels. Then, an additional 150 nm thick Au layer was deposited using the sputtering method to form source-drain contact electrodes. The length and the width of the channel were set to 200 μm and 400 μm, respectively. The SiO_2_ served as the back-gate dielectric and the heavily doped Si substrate as the back-gate. The e-FET devices were fabricated by transferring the 1:2 molar ratio nanomesh onto interdigitated Au electrodes (Dropsens, IDEAU200). These electrodes served as the source and drain electrodes of the e-FET. The electrode gap (channel length) was 200 μm. The surface of the gold electrodes that was not covered by the nanomesh was passivated using cyanoacrylate adhesive. For the conjugation of the aptamers onto the nanomesh channel for the binding of bisphenol A (BPA), a specific aptamer^46^ with a modification of amine group at 5′ ends (NH_2_-C_6_-(T)_10_-CCGGTGGGTGGTCAGGTGGGATAGCGTTCCGCGTATGGCCCAGCGCATCACGGGTTCGCACCA, Bioneer Corporation, Daejeon, Korea) was synthesized and cross-linked onto the amine functional groups of the p8GB#1 phage surface using a cross-linker (1% glutaraldehyde). The device was carefully washed with DI water after cross-linking in order to remove physically absorbed aptamer molecules.

### FET and e-FET measurements

Back-gated FETs were characterized using a physical property measurement system (PPMS, Quantum design). For the e-FET measurement, the source, drain and the water-based electrolyte gate (WG) potential were driven in a biological liquid electrolyte (10 mM PBS) using a bipotentiostat system (Autolab, PGSTAT 128N). Briefly, the source and drain electrodes were designated as two working electrodes (WE1 and WE2), the Ag/AgCl reference electrode (RE) (~2 mm dia. filled with 3 M KCl) as the water-gated electrolyte gate electrode, and the additional Pt wire as the counter electrode (CE). The I_DS_-V_DS_ and I_DS_-V_WG_ measurements of the e-FET device were taken in a PBS buffer (10 mM) at a scan rate of 10 mV/s. The pH-induced gating of the semiconducting nanomesh-based e-FET was analyzed in 10 mM phosphate citrate buffer solution by testing various pH values (3 ~ 9) at a fixed V_DS_ of 0.4 V with a scan rate of 10 mV/s. Dopamine solutions were prepared by dissolving dopamine hydrochloride (Sigma Aldrich) in 10 mM PBS buffer solution, and the e-FET measurement (gate sweep from −0.4 to 0.4 V) was carried out at a fixed V_DS_ of 0.1 V to prevent electrochemical oxidation of dopamine molecules. Bisphenol A (BPA) solution was prepared by dissolving BPA in methanol to the concentration level of 100 ppm first and then diluted by PBS solution to various concentrations. The e-FET measurement (gate sweep from −0.4 to 0.4 V) was carried out at a fixed V_DS_ of 0.2 V.

## Additional Information

**How to cite this article**: Byeon, H.-H. *et al.* Biologically templated assembly of hybrid semiconducting nanomesh for high performance field effect transistors and sensors. *Sci. Rep.* **6**, 35591; doi: 10.1038/srep35591 (2016).

## Supplementary Material

Supplementary Information
