# Supplementary material for: Biologically templated assembly of hybrid semiconducting nanomesh for high performance field effect transistors and sensors
Source: Sci Rep. 2016 Oct 20;6:35591. doi: 10.1038/srep35591 (PMC5071876; doi:10.1038/srep35591)
Supplement: Supplementary Information [file srep35591-s1.pdf]

Supplementary information for

Biologically templated assembly of hybrid semiconducting nanomesh for high performance field effect transistors and sensors

Hye-Hyeon Byeon[1, 2+], Seung-Woo Lee[1+], Eun-Hee Lee[3], Woong Kim[4], and Hyunjung Yi[1*]*

[1]Post-Silicon Semiconductor Institute, Korea Institute of Science and Technology, Seoul, 02792, Republic of Korea

[2]Department of Nano Semiconductor Engineering, Korea University, Seoul, 02841, Republic of Korea

[3]Department of Environmental Science and Engineering, Ewha Womans University, Seoul, 03760, Republic of Korea

[4]Department of Materials Science and Engineering, Korea University, 02841, Republic of Korea

[+] These authors contributed equally to this work.

[*] Address correspondence to hjungyi@kist.re.kr, woongkim@korea.ac.kr.

Supplementary Figures

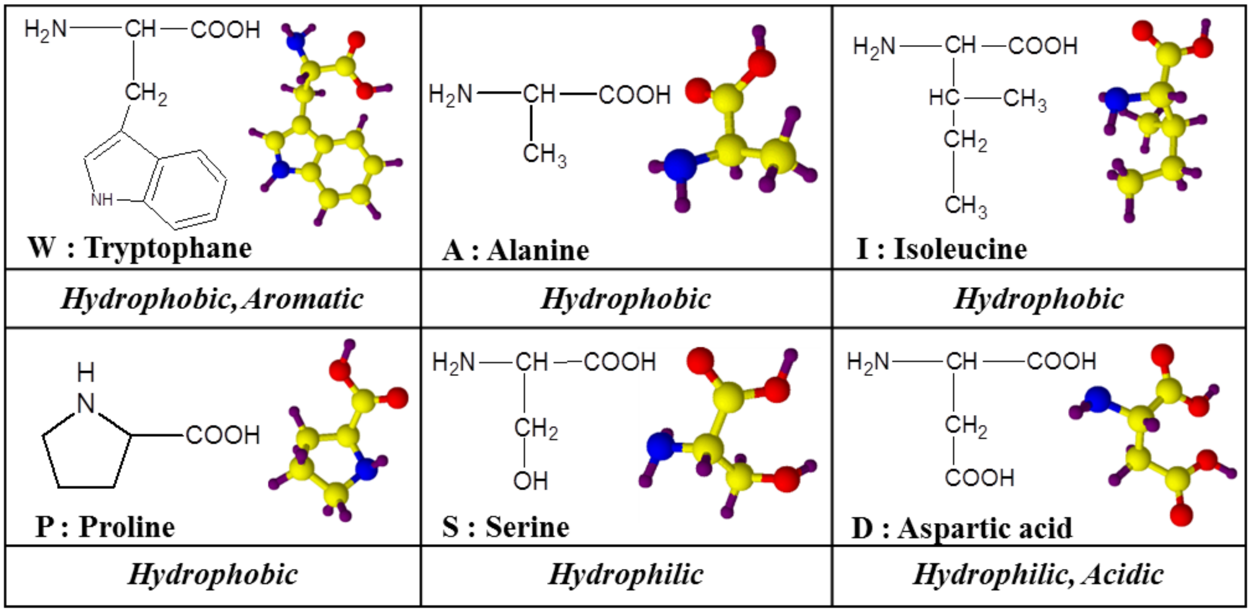

Figure S1. Representative amino acids of the p8 peptide of the p8GB#1 phage and its properties.

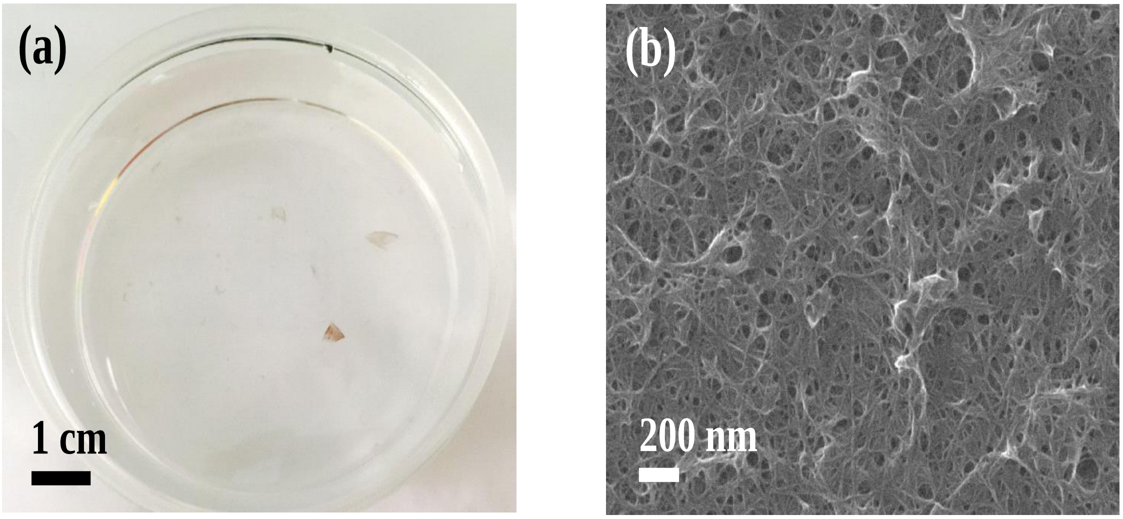

Figure S2. (a) Photograph of the control SWNT sample dialyzed in the absence of p8GB#1. (b) Scanning electron micrograph of the SWNTs dialyzed in the absence of p8GB#1.

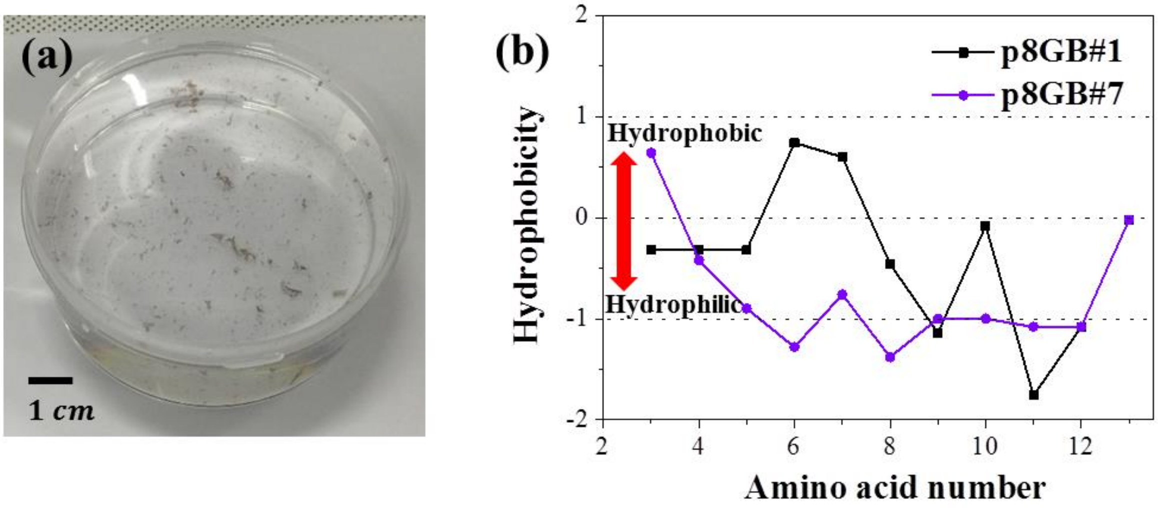

Figure S3. (a) Photograph of the semiconducting nanomesh assembled in the presence of another M13 phage clone (p8GB#7) without strong binding affinity toward SWNTs. The p8 peptide sequence of the p8GB#7 is VPSGQAEA. (b) SWNT:p8GB#7=2:1. The hydrophobicity plot was calculated using the Kyte-Doolittle scale and the window size of five. The molar ratio of SWNT:p8GB#7=2:1. A 1 × 10^{12}/mL number concentration of SWNTs was used to prepare a nanomesh with a 2:1 molar ratio of SWNT:p8GB#1.

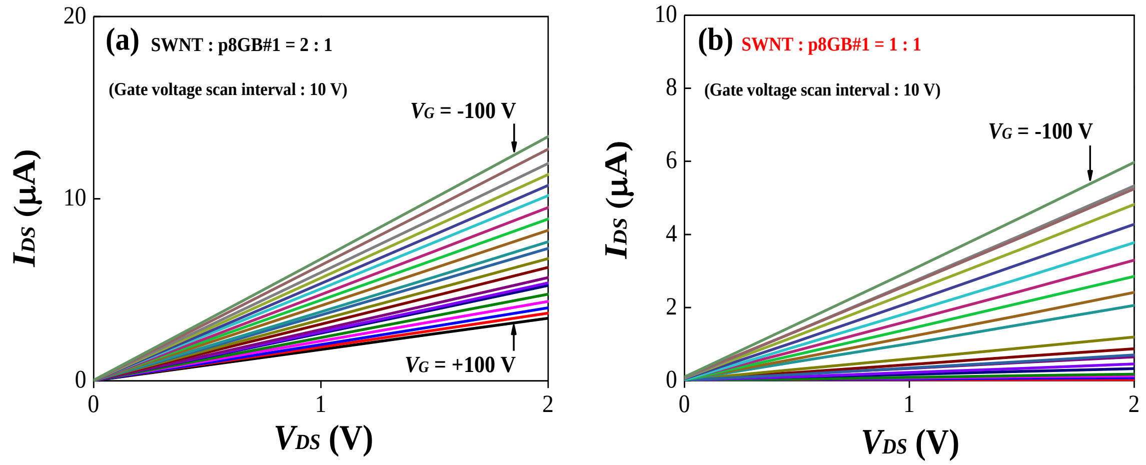

Figure S4. (a) Output characteristics $I_{DS} - V_{DS}$ in terms of V_G, for SWNT : p8GB#1 molar ratio of 2 : 1. (b) $I_{DS} - V_{DS}$ in terms of V_G, for SWNT : p8GB#1 molar ratio of 1 : 1.

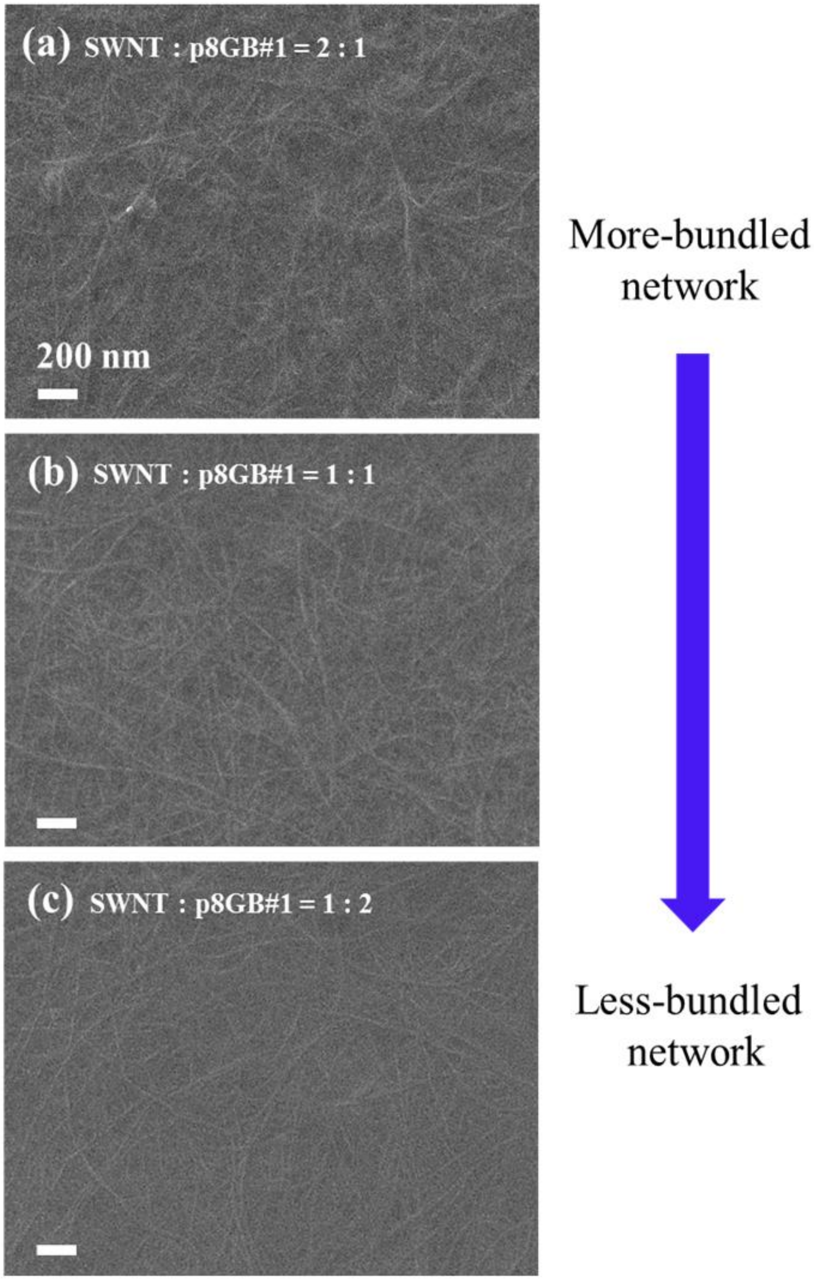

Figure S5. Field emission scanning electron micrographs of the hybrid nanomesh with various SWNT: p8GB#1 molar ratios: (a) SWNT: p8GB#1 = 2:1, (b) SWNT: p8GB#1 = 1:1, (c) SWNT: p8GB#1 = 1:2. The scale bar is 200 nm.

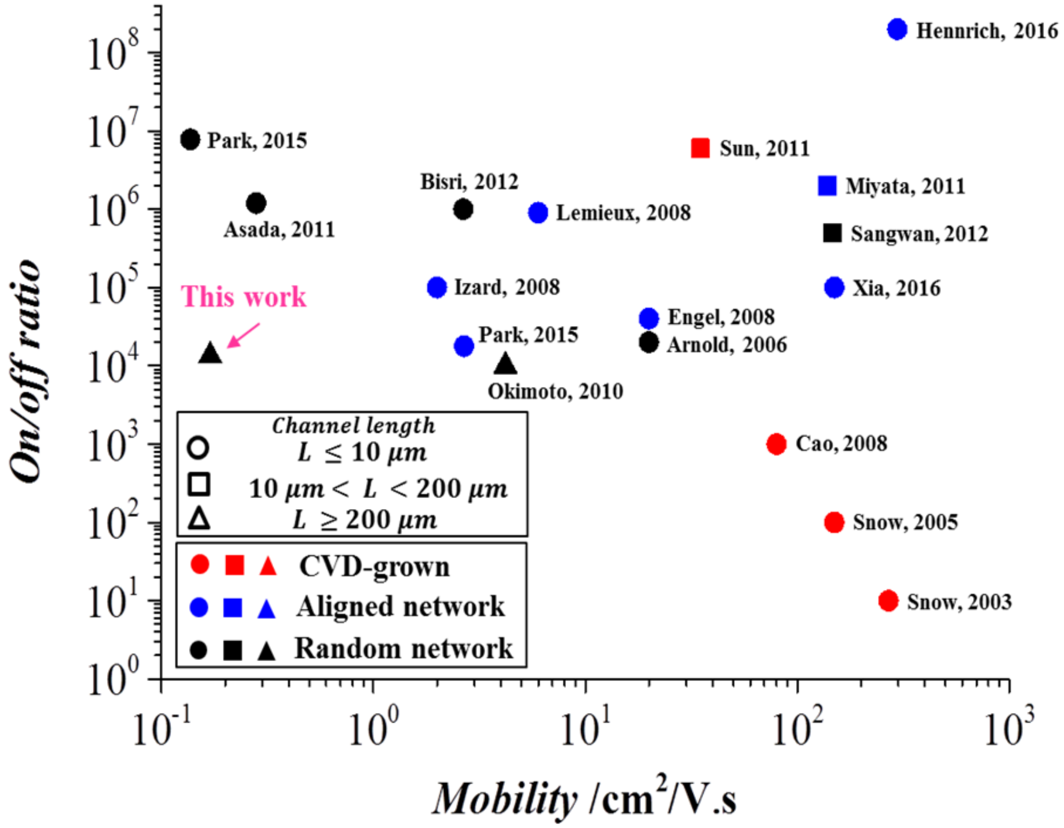

Figure S6. Comparison of the performance of the nanomesh-based FET reported in this work with other SWNT-based FETs fabricated by other approaches.[S1-S16]

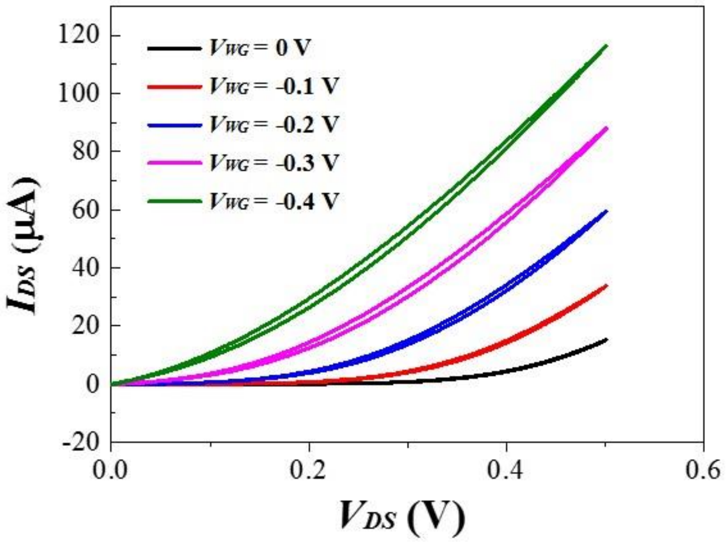

Figure S7. Output characteristics $I_{DS} - V_{DS}$ in terms of V_{WG}, for SWNT:p8GB#1 molar ratio of 1:2 in a buffer solution (PBS, 10 mM, pH=7.2).

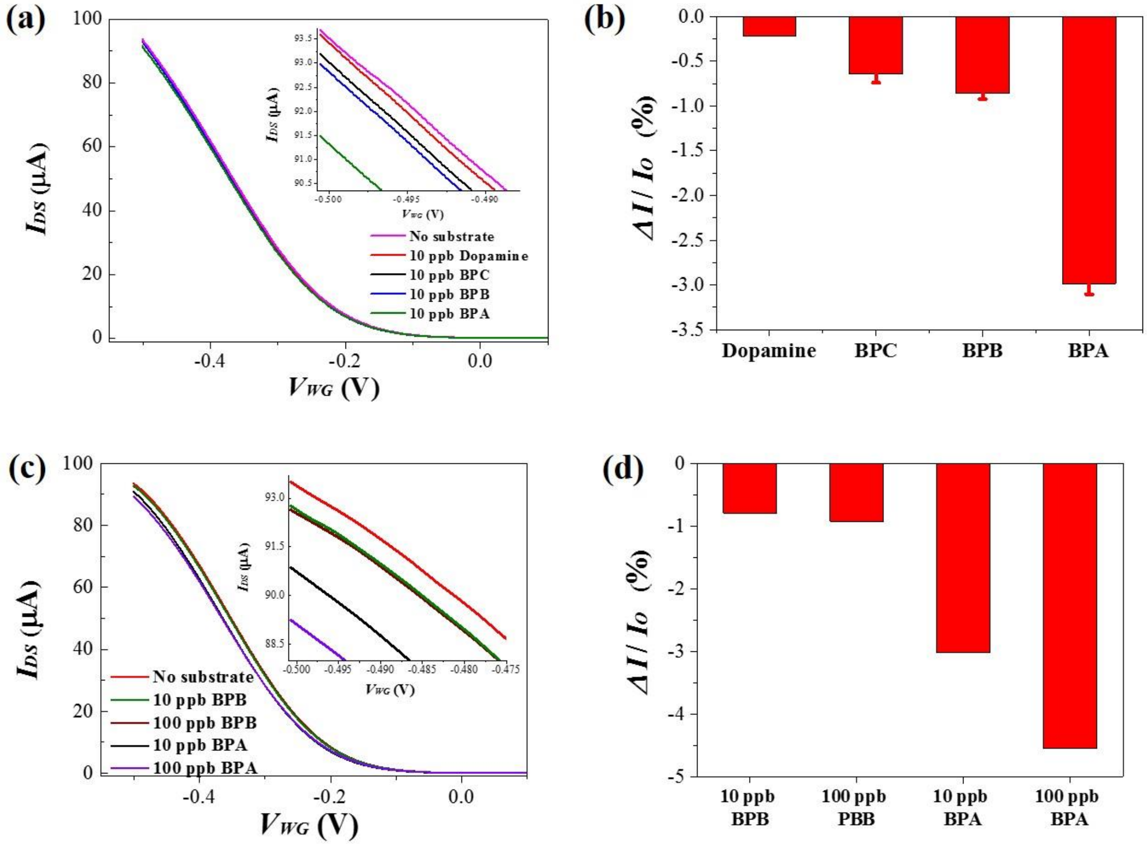

Figure S8. (a) The transfer characteristics of the anti-BPA aptamer-conjugated semiconducting nanomesh-based e-FET at various analytes in a biologically relevant solution (10 mM PBS buffer solution). The semiconducting nanomesh was prepared at a molar ratio of SWNT:p8GB#1=2:4. 'No substrate' is for the aptamer-conjugated e-FET only. (b) The comparison of the I_{DS} change upon the addition of various analytes. The anti-BPA aptamer-conjugated semiconducting nanomesh did not significantly respond to dopamine, or other BPA analogues such as bisphenol B (BPB) and bisphenol C (BPC) that are not expected to preferentially bind to the anti-BPA aptamer. Each data point is the mean±s.d. from n=2 except the dopamine. (c) The transfer characteristics of the anti-BPA aptamer-conjugated

semiconducting nanomesh-based e-FET at 10 ppb and 100 ppb of BPA and BPB. (d) The comparison of the I_{DS} change upon the addition of 10 ppb and 100 ppb of BPA and BPB. While 10 ppb and 100 ppb of BPB only exhibited ~ 1% change, the addition of 10 ppb and 100 ppb BPA caused ~ 3.1 and 4.4 % current changes, respectively, due to the specific binding of BPA to the aptamer.

Supplementary Equations

Calculation of the number concentration of SWNTs

We use as-received 99% semiconductor-enriched SWNT solution from NanoIntegris, Inc. The mean diameter and mean length of SWNTs were 1.4 nm and 1 μm, respectively. The CNT lattice constant is 1.42 Å $(1.42 \times 10^{-10}\ m)$. The concentration of SWNTs was calculated as follows:

Average area of one carbon atom (hexagonal structure of honey comb lattice):

$$\frac{\frac{3\sqrt{3}}{2} \times (1.42 \times 10^{-10} m)^2}{2} = \ 0.0262\ \mathrm{n}m^2$$

Average number of carbon per SWNTs:

$$\frac{2 \times \pi \times 0.7\ nm \times 1\ \mu m}{0.0262\ nm^2} = 1.68 \times 10^5$$

Average SWNT weight:

$$\frac{(1.68 \times 10^5) \times (\frac{12g}{mol})}{(6.02 \times \frac{10^{23}}{mol})} = 3.35 \times 10^{-18}\ g$$

The mass concentration of SWNTs (semiconductor 99%) was 0.01 mg/mL according to the production information sheet, and therefore SWNTs solution gave number concentration of ~3×10^{12} SWNTs/mL.

Calculation of the hole mobility of the back-gated nanomesh-FET

In the back-gated FET, the gate oxide capacitance ($C_{300\text{-nm thick SiO2}}$) and the SWNT capacitance (C_{SWNTs}) are connected in series. Therefore,

$$1/C_t=1/\ C_{300\text{-nm thick SiO2}} + 1/\ C_{SWNTs}$$

In this scheme, the lower capacitance dominates the total capacitance (C_t). The degree of the contribution of the gate oxide and the SWNTs to the total capacitance is determined by considering both the coverage of SWNTs (or network density) and the thickness of the gate oxide. The SWNT capacitance is known to increase with increasing coverage at a very low coverage range and then saturate at <~1% of coverage for a few hundred of nm-thick gate oxide.[S2, S16] In general, when the spacing between SWNTs becomes comparable to or smaller than the thickness of the gate oxide, then the gate capacitance is mostly dominated by the gate oxide. In our case, the thickness of the gate oxide is 300 nm, and the average spacing of SWNTs is much smaller than 300 nm according to the SEM image of the nanomesh. Therefore, we assumed that the gate capacitance would be mostly dominated by the gate oxide. Thus, the field-effect hole mobility of the FET was calculated using the parallel plate model:

$$\mu_h = [(\Delta I_{DS}/V_{DS})(L/W)]/Cox\Delta V_G$$

where W (400 μm) and L (200 μm) are channel width and length, respectively, and C_{ox} is the gate oxide capacitance per unit area which is 1.15 × 10-8 F/cm^2 for the 300 nm-thick SiO_2. I_{DS}, V_{DS} and V_G are the source-drain current, the source-drain voltage and the gate voltage,

respectively, and the data were extracted from the measured transfer characteristics in the linear region. Strictly speaking, this assumption slightly overestimates the real capacitance and therefore slightly underestimates the mobility.

References

S1 Sun, D. *et al*. Flexible High-Performance Carbon Nanotube Integrated Circuits. *Nat. Nanotechnol.* **6**, 156-161 (2011).

S2 Snow, E. S., Campbell, P. M. & Ancona, M. G. High-Mobility Carbon-Nanotube Thin-Film Transistors on a Polymeric Substrate. *Appl. Phys. Lett.* **86**, 033105 (2005).

S3 Snow, E. S., Novak J. P., Campbell. P. M. & Park. D. Random Networks of Carbon Nanotubes As an Electronic Material. *Appl. Phys. Lett.* **82**, 2145-2147 (2003).

S4 Cao, Q. *et al*. Medium-Scale Carbon Nanotube Thin-Film Integrated Circuits on Flexible Plastic Substrates. *Nature* **454**, 495-500 (2008).

S5 Izard, N. *et al*. Semiconductor-Enriched Single Wall Carbon Nanotube Networks Applied to Field Effect Transistors. *Appl. Phys. Lett.* **92**, 243112 (2008).

S6 Miyata, Y. *et al*. Length-Sorted Semiconducting Carbon Nanotubes for High-Mobility Thin Film Transistors. *Nano Res.* **4**, 963-970 (2011).

S7 Lemieux, M. C. *et al*. Self-Sorted, Aligned Nanotube Networks for Thin-Film Transistors.

Science **321**, 101-104 (2008).

S8 Engel, M. *et al*. Thin Film Nanotube Transistors Based on Self-Assembled, Aligned, Semiconducting Carbon Nanotube Arrays. *ACS Nano* **2**, 2445-2452 (2008).

S9 Park, S. *et al*. Large-Area Assembly of Densely Aligned Single-Walled Carbon Nanotubes Using Solution Shearing and Their Application to Field-Effect Transistors. *Adv. Mater.* **27**, 2656-2662 (2015).

S10 Hennrich, F. *et al*. Length-Sorted, Large-Diameter, Polyfluorene-Wrapped Semiconducting Single-Walled Carbon Nanotubes for High-Density, Short-Channel Transistors. *ACS Nano* **10**, 1888-1895 (2016).

S11 Xia, J. *et al*. Metal Contact Effect on the Performance and Scaling Behavior of Carbon Nanotube Thin Film Transistors. *Nanoscale* **8**, 9988-9996 (2016).

S12 Arnold, M. S. *et al*. Sorting Carbon Nanotubes by Electronic Structure Using Density Differentiation. *Nat. Nanotechnol.* **1**, 60-65 (2006).

S13 Bisri, S. Z. *et al*. High Performance Ambipolar Field-Effect Transistor of Random Network Carbon Nanotubes. *Adv. Mater.* **24**, 6147-6152 (2012).

S14 Sangwan, V. K. *et al*. Fundamental Performance Limits of Carbon Nanotube Thin-Film Transistors Achieved Using Hybrid Molecular Dielectrics. *ACS Nano* **6**, 7480-7488 (2012).

S15 Asada, Y. *et al*. Thin-Film Transistors with Length-Sorted DNA-Wrapped Single-Walled Carbon Nanotubes. *J. Phys. Chem. C* **115**, 270-273 (2011).

S16 Okimoto, H. *et al*. Tunable Carbon Nanotube Thin-Film Transistors Produced Exclusively Via Inkjet Printing. *Adv. Mater.* **22**, 3981-3986 (2010).
